# Cognitive bias modification to prevent depression (COPE): study protocol for a randomised controlled trial

**DOI:** 10.1186/1745-6215-15-282

**Published:** 2014-07-11

**Authors:** Osvaldo P Almeida, Colin MacLeod, Andrew Ford, Ben Grafton, Varsha Hirani, David Glance, Emily Holmes

**Affiliations:** 1 Western Australian Centre for Health & Ageing (M573), Centre for Medical Research of the Perkins Institute for Medical Research, University of Western Australia, 35 Stirling Highway, Crawley, WA 6009, Australia; 2 School of Psychiatry & Clinical Neurosciences, University of Western Australia, 35 Stirling Highway, Crawley, WA 6009, Australia; 3 Department of Psychiatry, Royal Perth Hospital, Level 6, Ainslie House, 48 Murray Street, PerthWA 6000, Australia; 4 School of Psychology, University of Western Australia, 35 Stirling Highway, Crawley, WA 6009, Australia; 5 Centre for Software Practice, University of Western Australia, 35 Stirling Highway, Crawley, WA 6009, Australia; 6 MRC Cognition and Brain Sciences Unit, 15 Chaucer Road, Cambridge CB2 7EF, UK

**Keywords:** Anxiety, Cognitive bias modification, Depression, Depressive disorder, Indicated prevention, Prevention, Randomised controlled trial

## Abstract

**Background:**

Depression is a leading cause of disability worldwide and, although efficacious treatments are available, their efficacy is suboptimal and recurrence of symptoms is common. Effective preventive strategies could reduce disability and the long term social and health complications associated with the disorder, but current options are limited. Cognitive bias modification (CBM) is a novel, simple, and safe intervention that addresses attentional and interpretive biases associated with anxiety, dysphoria, and depression. The primary aim of this trial is to determine if CBM decreases the one-year onset of a major depressive episode among adults with subsyndromal depression.

**Design and methods:**

This randomised controlled trial will recruit 532 adults with subsyndromal symptoms of depression living in the Australian community (parallel design, 1:1 allocation ratio). Participants will be free of clinically significant symptoms of depression and of psychotic disorders, sensory and cognitive impairment, and risky alcohol use. The CBM intervention will target attentional and interpretive biases associated with depressive symptoms. The sessions will be delivered via the internet over a period of 52 weeks. The primary outcome of interest is the onset of a major depressive episode according the DSM-IV-TR criteria over a 12-month period. Secondary outcomes of interest include change in the severity of depressive symptoms as measured by the Patient Health Questionnaire (PHQ-9), use of antidepressants or benzodiazepines, and changes in attention and interpretive biases. The assessment of outcomes will take place 3, 6, 9, and 12 months after randomisation and will occur via the internet.

**Discussion:**

We propose to test the efficacy of an innovative intervention that is well grounded in theory and for which increasing empirical evidence for an effect on mood is available. The intervention is simple, inexpensive, easy to access, and could be easily rolled out into practice if our findings confirm a role for CBM in the prevention of depression.

**Trial registration:**

Australian and New Zealand Clinical Trials Registry ACTRN12613001334796. Date: 5th December 2013.

## Background

Depression affects people of all ages and has an estimated lifetime prevalence of 27% [1]. The personal and social costs associated with depression are difficult to measure, but currently available evidence indicates that direct financial loss is in the order of A$100,000 over the lifetime of a person and approximately A$15 billion per annum for the Australian society [2]. People with depression have more unplanned admissions to general hospitals and longer length of stay than people without depression and, once admitted, they have nearly twice the risk of death as those without past history of depression [3]. In other words, depression is a common, costly, and disabling disorder that reduces life expectancy. Consequently, the successful prevention of depression would lead to important health and socioeconomic benefits.

### Currently available strategies to prevent depression: who should we target?

Various approaches have been used with the aim of reducing the prevalence of depression in the community. Some have focused on improving the identification of people with depression, while others have sought to increase the efficacy and effectiveness of existing management strategies. A meta-analysis of 16 randomised trials has shown that the systematic use of screening instruments or case-finding procedures increases the recognition of depression by a modest 27%, and this has no effect on the adoption of treatments or the clinical outcome of patients [4]. Similarly, the evidence to support the value of educational interventions targeting medical practitioners to enhance the treatment of depression is not robust [5]. In view of these limitations, increasing emphasis has been placed on the potential value of strategies that instead seek to reduce the prevalence of depression through prevention.

Recent pragmatic approaches to prevent depression have focused on the targeted delivery of programs to people with symptoms of depression that do not reach threshold for the diagnosis of a disorder, an approach known as indicated prevention [6]. The rationale, in this case, is that people with ‘subsyndromal depression’ are at greater risk of developing a full-blown major depressive episode than people who are free of depressive symptoms, and so may benefit most from interventions designed to reduce risk. Data from the PROSPECT trial, which recruited older adults from primary care practices in New York, Philadelphia, and Pittsburgh, showed that 10.4% of people with subsyndromal depression developed a major depressive episode within one year compared with 0.8% of participants who were free of these symptoms [7]. The PIKO study is a good illustration of a trial of indicated prevention [8], where the investigators recruited 170 adults aged 75 years or over who scored 16 or more on the Center for Epidemiologic Studies Depression Scale (CES-D) but did not fulfil DSM-IV criteria for a depressive or anxiety disorder at the time of assessment or during the preceding 12 months. They implemented a stepped care intervention that consisted of ‘watchful waiting’ for the first 3 months, then potentially progressing to bibliotherapy (if subsequent CES-D ≥16), followed by problem solving therapy and, lastly, treatment with antidepressant medications. The relative risk of developing a depressive or anxiety disorder among people receiving the intervention compared with usual care was 0.49 (95% confidence interval = 0.24–0.98) by the end of 12 months. During this period, 4 participants met criteria for a depressive disorder, 3 for an anxiety disorder, and 2 for mixed anxiety and depression in the intervention group compared with 10 cases of depressive disorder, 5 of anxiety, and 5 mixed states in the usual care group. However, there was no statistically significant difference between the groups when the outcome of the study was limited to depression. In addition, dropout was higher in the intervention than in the usual care group (24/86 vs. 8/84), suggesting problems with the acceptability of the intervention by participants.

The value of indicated prevention approaches in reducing the risk of major depression is not restricted to older adults. A recent review of studies across the lifespan reported data on 33 trials of indicated or selective prevention (for example, post-stroke patients), most of which used cognitive behavioural therapy (CBT) or problem solving as the active intervention [9]. There was an overall benefit of the interventions (about 25% relative risk reduction of clinically significant depression), although the non-independence of some studies was not taken into account in the analyses (i.e., trials with more than one intervention group were treated as separate studies).

In summary, existing evidence indicates that adults with subsyndromal symptoms of depression have substantially greater risk of developing a major depressive episode over the subsequent year compared with people free of these symptoms. Averting the onset of major depression in people at risk seems feasible, but clearly effective preventive strategies are yet to be established.

### Biased thinking patterns are both markers and predictors of depression

The theoretical framework underpinning CBT posits that depressed mood is produced by a set of biased thinking processes that commonly involve preferential attention to negative aspects of experience and an inflated tendency to attribute negative interpretations to ambiguous events. Such a bias in the processing of information initiates the cycle of negative thinking, dysfunctional beliefs, withdrawal, and psychomotor changes that are characteristic of depression [10]. CBT challenges negative thoughts about the self, others, and the future, by teaching patients to rationally appraise these thoughts and to generate more accurate and functional alternatives. Effective shifts of dysfunctional biased beliefs have been associated with a robust response to treatment and a decreased risk of relapse of symptoms [11]. Importantly, such a pattern of biased beliefs and negative thinking are strong predictors of the development of future depression. For example, children with no history of depression, but known to be at high risk on the basis of a parent’s history, exhibit the same biased pattern of negative thinking and beliefs [12]. In a longitudinal study carried out over a 2.5 year period with 347 college students, those who initially displayed negatively biased thinking were 3 to 7 times as likely as controls to develop an episode of major depression [13]. These findings indicate that if the mechanisms that give rise to negatively biased thoughts and beliefs could be identified and effectively targeted, this could protect against the future development of depression in high-risk individuals.

Emerging empirical and clinical findings are consistent with the hypothesis that selective biases in attention and interpretation, operating to favour the processing of emotionally negative information, represent the psychological basis of disordered mood [14-16]. For example, adults with mood disorders shown a series of faces on a computer screen selectively direct their attention to sad faces, but show no such a bias when presented with angry or happy faces [17]. Moreover, people without a history of depression, but known to be at heightened risk (e.g., daughters of depressed mothers), display this same attentional bias to negative information [18]. Such findings are consistent with the idea that attentional bias precedes and potentially contributes to the precipitation of depressive episodes. In addition, when faced with ambiguity, people with depressed mood favour negative interpretations of stimuli. For example, when presented the ambiguous cue word ‘GROWTH’, people with low mood are faster to then complete fragments of target words semantically related to the negative rather than the non-negative meanings of the ambiguous cue: C_NC_ER and GR_AT_R (cancer and greater) [19]. Once again, such bias has been found to characterise people with no history of depression known to be at high risk of developing this disorder [20]. These results confirm that attentional and interpretation biases are not only associated with concurrent disturbances of mood, but also represent risk factors for future mood disorders. They also invite the question: are these biases amenable to change?

### Cognitive bias modification (CBM) procedures are effective at extinguishing selective processing bias

MacLeod and colleagues introduced the most widely used approach worldwide to modify attentional bias [21]. The procedure exposes participants to pairs of words or images on a computer screen for 500 ms, with each pair including one emotionally negative and one neutral item. Immediately after the words/images disappear, a single small visual probe (vertical or horizontal white line) is presented in the same spatial position where one of the original stimuli had been displayed. Participants are required to indicate, as quickly as possible, the orientation of this probe (horizontal or vertical), and their speed to accurately do so is recorded over dozens of trials. People who display an attentional bias to the more negative information are faster to make discrimination judgments for probes that appear in the same area as the negative stimuli, compared to probes in the area of the neutral stimuli [21]. Cognitive bias modification for attention (CBM-A) delivers hundreds to thousands of trials in which all probes are consistently presented where the neutral rather than the negative stimuli had just appeared, in order to encourage development of an attentional avoidance of negative response to information. A control condition presents probes with equal frequency to each of these two areas. In CBM designed to reduce negative interpretations (CBM-I), participants are exposed to trials that present ambiguous information, followed by a target word fragment that must be completed in a semantically consistent manner. CBM-I delivers hundreds of trials in which target fragments can yield only words consistent with non-negative interpretations of the ambiguity, thus encouraging the tendency to interpret ambiguity in a benign manner. Hence, the ambiguous text “When you chat to people at a party they are soon chuckling, because you are so”, will be followed by the fragment W_T_Y (yielding WITTY). A control condition employs fragments that equally often yield words consistent with negative (e.g., S_L_Y) or non-negative interpretation of the ambiguity.

### CBM reduces anxiety and improves mood

Emerging research shows that CBM-A attenuates anxiety reactions to stressful life events [22], reduces recurrent negative thought intrusions in chronic worriers [23], decreases avoidant behaviours [24], and mitigates the intensity of depressive symptoms among dysphoric patients over a 2-week period [25]. However, negative findings have also been reported, with an internet-based intervention failing to improve symptoms associated with social anxiety over a period of 4 weeks [26]. A recent, as yet unpublished, meta-analysis concluded that CBM is associated with small beneficial effects on symptoms of anxiety and depression, but that the quality and size of the trials published to date is suboptimal (personal communication). In addition, CBM-A normalises the cortisol awakening response that is characteristic of depressive disorders [27]. The results of CBM-I interventions indicate that they too reduce negative emotions following stress [28,29]. There is preliminary evidence that combining CBM-A and CBM-I may be useful in reducing existing emotional dysfunction. For example, Beard and colleagues randomly assigned 32 adults with social anxiety disorder to 16 sessions of combined CBM over a period of 8 weeks: participants exposed to this combined CBM experienced a significantly greater reduction of anxiety symptoms than controls [30]. However, it remains to be established if this combination of CBM-A and CBM-I can reduce the risk of a novel episode of major depression when delivered to participants known to be at increased risk for the disorder.

The primary aim of this trial is to determine if CBM designed to attenuate negative attentional and interpretive biases decreases the one-year onset of major depressive episodes among adults with subsyndromal symptoms of depression. The primary hypothesis of this trial is that a lower proportion of participants assigned active compared with control CBM will develop a depressive episode over a period of one year. The study will also test whether, compared with the control group, CBM reduces the severity of depressive symptoms and lowers the frequency of antidepressant and benzodiazepine use, as well as reducing negative attentional and interpretive biases.

## Methods

### Trial design

The COgnitive bias modification to Prevent dEpression (COPE) study is a randomised, double-blind, parallel, controlled trial of CBM delivered over the internet. The allocation ratio will be 1:1.

### Ethics

The activities and procedures of this trial follow the principles outlined by the Declaration of Helsinki. The Human Research Ethics Committee of the Department of Health of Western Australia has approved the research protocol (#2013/57) and all participants will provide written informed consent.

### Participants and setting

We will recruit 532 adults with subsyndromal symptoms of depression living in the Western Australian community. They will need to fulfil the following criteria for participation:

– Patient Health Questionnaire (PHQ-9) total score between 5 and 14, inclusive (described below)

– Age ≥45 years (to avoid including people with bipolar disorder) [31]

– Fluent in written and spoken English

– Have access to a computer with internet connection

– No current or past DSM-IV-TR diagnosis of major depressive episode (Structured Clinical Interview for DSM Disorders (SCID-I) – see below)

– No current or past diagnosis of schizophrenia, schizoaffective disorder, or bipolar disorder

– No past history of stroke or of neurodegenerative diseases (e.g., Parkinson’s disease)

– Free of diseases likely to undermine ongoing participation in the study for 12 months (e.g., metastatic cancer)

– No evidence of alcohol abuse or dependence (AUDIT ≥15 – see below)

– No evidence of cognitive impairment (Telephone Interview for Cognitive Status (TICSm) <27 – see below)

– No evidence of active suicidal intent

– No evidence of visual impairment that might compromise ability to read or use the computer

– Registered with a general practitioner

We will obtain a random extraction of 20,000 names of adults aged 45 years or over living in the community from the Australian Electoral Commission (enrolment to vote is compulsory in Australia). They will receive, by post, information about the study together with a screening questionnaire designed to ascertain eligibility. The screening questionnaire will ascertain the following information: personal contact details, contact details of GP, gender, date of birth, date of assessment, place of birth, language spoken at home, self-perceived fluency in English, regular access and use of a computer and the internet, past diagnosis of neurological disorders (stroke, Parkinson’s disease, Alzheimer’s disease), current history of metastatic cancer, past diagnosis of depression, partial or total blindness, and list of all medications that the volunteer is currently using. The questionnaire will also include the following scales:

– PHQ-9: the PHQ-9 is a widely used and well-accepted screening instrument for depression [32,33]. The scale consists of 9 questions about how often the respondent has been bothered by depressive symptoms during the past 2 weeks, and each item can be scored 0 (not at all), 1 (several days), 2 (a week or more) or 3 (nearly every day). The 9 items are: (a) decreased interest or pleasure, (b) low mood, (c) sleep disturbance, (d) lack of energy, (e) disturbed appetite, (f) feelings of failure or guilt, (g) poor concentration, (h) psychomotor disturbance, and (i) suicidal thoughts. The PHQ-9 is a simple self-rating instrument that takes no longer than 5 minutes to complete. Scores between 5 and 14, inclusive, indicate mild or subsyndromal depression.

– Alcohol Use Disorders Identification Test (AUDIT): this 10-item questionnaire has been designed as a screening instrument for risk drinking. The scale takes 2 minutes to complete, and scores ≥15 indicate hazardous/harmful alcohol use or dependency [34].

Returned screening questionnaires will be immediately scanned and volunteers deemed potentially eligible will be contacted within 2 weeks to organise a telephone interview for the assessment of cognitive function (TICSm) [35] and, for those free of cognitive impairment, an assessment of current and past mental state with the SCID-I, which is a structured clinical interview for axis I diagnoses that can be administered by trained research staff over the telephone [36]. It provides reliable and valid information to establish the diagnosis of major depressive episode (recurrent or single episode) according to DSM-IV-TR criteria [37] (which is consistent with DSM-5 criteria). Participants with a current major depressive episode will be excluded from further participation. Volunteers deemed eligible will be given a user name and password to access the CBM website. When they first login, their username will be linked to a number from a random list of study numbers, which will be randomly assigned to either the intervention or control CBM. Additional lists will be requested from the Australian Electoral Commission until the total number of participants enrolled in the study reaches the required number of 532 people.

### Intervention

Eligible participants will be randomly allocated to one of two treatment arms: control or active CBM (parallel design, 1:1 allocation). CBM sessions will be delivered over a period of 52 weeks (1 year) according to the following schedule:

– three times per week for the first 4 weeks

– twice weekly for the next 4 weeks (up to week 8)

– once weekly for the next 18 weeks (up to week 26)

– once every other week for the next 12 weeks (up to week 38)

– once per month for the remainder of the follow-up period (up to week 52)

This delivery schedule is designed to achieve rapid bias modification through initially intensive training, while also encouraging maintenance of the resulting cognitive change by sustained exposure to CBM across an extended period and by phasing out rather than abruptly terminating the intervention. Each CBM session will last 20 minutes (10 minutes attention, 10 minutes interpretation), and will be accessed through a password-protected website. On each session, participants will be presented with a pair of emotionally-toned facial images (sad vs. neutral or happy) for 500 ms, which will then be replaced by a small white probe (vertical or horizontal line), appearing in the screen position previously occupied by one of these stimuli. Participants will be instructed to use their keyboard to indicate the orientation of this probe as quickly as possible (‘V’ for vertical, ‘H’ for horizontal). The probe will then disappear and will be replaced, after 1 second, by another stimulus pair that will commence the next trial. The time to discriminate probe identity will be recorded automatically. In the active CBM condition, all probes will appear in the opposite screen area from that of the negative stimulus, while in the control CBM condition probes will appear equally often in the area of the negative and non-negative stimuli.

The second half of each 20-minute session will deliver CBM-I using single words and short textual scenarios as ambiguous stimuli. On each trial, this ambiguous stimulus will first be presented (e.g., word ‘HIT’), followed, 500 ms later, by a fragment of a word that is semantically consistent with one or other meaning of the preceding ambiguity (e.g., ‘S_CCESS’ or ‘P_NCH’). Participants will be instructed to use their keyboard to complete this fragment to yield a word consistent with the meaning of the initial word or sentence. The time to solve the word fragments will be recorded automatically. In the active CBM condition all fragments will yield only words consistent with non-negative interpretations of the preceding ambiguity, while in the control CBM condition fragments will equally often yield words consistent with the negative and non-negative interpretation of this ambiguity.

### Outcomes and study measures

The primary outcome of interest of this study is the onset of a major depressive episode over a 12-month period, which we will assess with the SCID-I 3, 6, 9, and 12 months after randomisation. The use of the SCID-I will allow for the ascertainment of depressive episodes that may have occurred between assessments, and will also enable us to assess clinically significant symptoms of anxiety that may have arisen in isolation or comorbid with depression. This assessment will take place via a telephone interview.

Secondary outcomes of interest include: i) proportion of people experiencing the onset of clinically significant symptoms of depression, as established by a PHQ-9 total score of 15 or greater, ii) change in the severity of depressive symptoms as measured by the PHQ-9, iii) the proportion of people using antidepressants or benzodiazepines, and iv) changes in attention and interpretive biases. The assessment of these secondary outcomes will take place 3, 6, 9, and 12 months after randomisation and will occur via the internet (i.e., electronic questionnaire and assessment of cognitive bias using the same procedures described for the intervention).

Attentional bias will be measured as the difference, in ms, to respond to the probes appearing in location associated with the negative compared with the emotionally non-negative faces. Similarly, negative interpretive bias will be operationalised as the relative speeding to complete the words associated with the negative compared with the non-negative ambiguous words/contexts. Table 1 shows COPE’s assessment schedule.

**Table 1:** List and timeline of assessments for the Healthy Transition Trial

		**Month**				
**Assessments:**	**Screening**	**0**	**3**	**6**	**9**	**12**
Sociodemographic/clinical information (postal questionnaire)	YES	–	–	–	–	–
AUDIT (postal questionnaire)	YES	–	–	–	–	–
TICSm (telephone)	YES	–	–	–	–	–
SCID (telephone)	YES	–	YES	YES	YES	YES
PHQ-9 (postal for screening; internet for all other endpoints)*	YES	–	YES	YES	YES	YES
Attentional and interpretive bias	–	YES	YES	YES	YES	YES

*Includes an additional 6-week assessment. AUDIT, Alcohol use disorders identification test; PHQ-9, Patient Health Questionnaire; SCID, Structured Clinical Interview for DSM Disorders; TICSm, Telephone interview for cognitive status.

None of the activities or procedures of COPE are hazardous. Adherence to the study protocol will be recoded automatically through monitoring of scheduled activities: 70 to 100% compliant, 50 to 70% partly compliant, <50% non-compliant. Participants who drop out from the study will be contacted by telephone to ascertain the reasons for their discontinuation, which will be recorded and later reported.

### Data entry, coding, security, and storage

The contact details of consenting participants will be kept in an Excel spreadsheet that will also contain a unique identifier number. This unique identifier will be generated by computer and will be used to identify participants in the study working database. Data collected via the postal questionnaire and interview with the SCID will be entered manually into an Excel spreadsheet. Data collected via the internet will be recorded automatically and later transported to an Excel spreadsheet, which will then be imported into Stata for statistical analysis (StataCorp, version 13.1). Postal and SCID data will be kept in a locked filing cabinet at the Western Australia Centre for Health & Ageing. Electronic data will be kept in a secure server of the University of Western Australia. Study data will be kept for a minimum of 7 years following completion of the trial.

### Sample size

About 10% of adults with subsyndromal symptoms of depression develop a major depressive episode over the subsequent 12 months [7]. We anticipate that CBM will be associated with an absolute risk reduction of major depression of 6% over this period (i.e., annual incidence of 4% compared with 0.8% for people free of subsyndromal depression – still high, but lower than controls with subsyndromal depression). This would require that 444 (222 per group) participants provide valid data to ascertain this endpoint of the study (power of 80% and two-tailed alpha of 5%). We further anticipate that as many as 20% of our volunteers may be lost during follow-up, which would require that 532 participants enter the study (266 per group). The number need to treat for one to benefit from CBM would be 17. If the PHQ-9 scores of participants declines, on average, 0.5 points for controls (SD = 4) and 3 points for active CBM participants (SD = 1.5), then a study of this size would have 97% power to declare such difference between the groups as statistically significant (two-sided alpha = 5%). The changes in response bias (measured in ms) are expected to be associated with a minimum effect size of 0.4 (Cohen’s d) [22], which would require about 50 trials per participant (within group comparison). As each CBM session includes 180 trials for attention (faces) and 180 trials for interpretive bias (words), the study will have ample power to investigate this outcome.

Two of our previous community-based trials showed that 19.8% of mature adults have subsyndromal depressive symptoms [5,38], indicating that we will need to screen 2,687 community-dwelling people to accrue 532 eligible cases. Response to our previous invitations has been of the order of 15 to 20%. If we assume that 15% of those invited will consent to screening, we will need to invite 18,000 people, to screen 2,687 participants, and recruit 532 into the trial. We will seek to approach 20,000 people.

### Randomisation and blinding

Participants will be assigned to one of the two treatment arms of the study according to lists of random numbers generated by computer in random permuted blocks of 8 to 20. The lists will be generated and maintained by the Centre for Software Practice of the University of Western Australia, which will have no direct contact with participants. Neither staff nor participants will be aware of group assignment. Participants will be instructed that the focus of their activity is to identify the orientation of the probe (vertical or horizontal) and the missing letter of the word fragment as quickly as possible. In this respect, control and active CBM tasks are indistinguishable. Research staff involved in the collection of endpoints will be directed not to discuss with participants any aspects of the intervention. Unblinding will only occur once the final endpoint of interest is collected from the last participant in the trial.

All logins and logouts will be recorded automatically, so that the trial will have an objective measure of the adherence of participants to the scheduled activities. In order to optimise the completion of scheduled activities of the study, participants will receive an automated text message and/or email (as per stated preference) alerting them to upcoming study activities for the week ahead and encouraging them to complete the relevant tasks. Once an activity is completed, participants will receive a text/email thanking them and outlining the next activity in their schedule.

### Statistical methods

We will use means and standard deviations to describe continuous variables with normal distribution, medians, and inter-quartile ranges for ranked variables, and frequency tables for categorical variables. We will evaluate the primary endpoint of the study by considering the proportion of participants showing evidence of depression during follow-up. Our approach to the analysis will be intention-to-treat and will use panel data accrued at different time points. We will use the command ‘xtlogit’ of the statistical package Stata 13.1 to complete these analyses, and models will be adjusted for imbalances that might be present at baseline. This approach to the analysis will also allow us to investigate interactions between treatment group and time. Similarly, we will use multilevel mixed models to analyse changes in PHQ-9 scores and in attentional and interpretive biases from baseline to month 12. We will investigate the interaction between group and time effects (statistical adjustments will be made, if necessary). This analysis will also be intention-to-treat. This approach to the analysis of the data (panel; repeated measures) increases study power and lessens the impact of missing data. All probability tests will be two-tailed (*P* <0.05).

## Discussion

Depression is a leading cause of disability and health expenditure in Australia and worldwide. Attempts to develop strategies to prevent depression have focused predominantly on people at high risk, such as those with subsyndromal symptoms, although results have been mixed and costs high. COPE will test the efficacy of a highly innovative intervention that is well grounded in theory and for which increasing empirical evidence for an effect on mood is available. We used best available evidence to estimate the number of people with subsyndromal symptoms of depression likely to develop a depressive episode over a 1-year period [7], and a schedule of activities designed to shift existing biases during the first few weeks of treatment and progressively less frequent booster sessions designed to sustain these changes. The intervention targets both attentional and interpretative biases, as this seems to enhance treatment response [30]. This intervention is simple, inexpensive, easy to access via the internet, and could be easily rolled out into practice if our findings confirm a role for CBM in the prevention of depression. Our strategy to recruit participants has been used successfully before and will give us the flexibility to expand recruitment in a timely manner, if necessary. All study procedures have been piloted: they are working as planned and have been well accepted by users. We anticipate that we will need three years to complete the collection of all outcomes of this study.

## Trial status

The COPE trial is currently recruiting participants.

## Abbreviations

AUDIT: Alcohol use disorders identification test; CBM: Cognitive bias modification; CBM-A: CBM of attention; CBM-I: CBM of interpretation; CBT: Cognitive behavioural therapy; CES-D: Center for Epidemiologic Studies Depression Scale; COPE: COgnitive bias modification to Prevent dEpression; PHQ-9: Patient Health Questionnaire; SCID-I: Structured clinical interview for DSM Disorders, axis I; TICSm: Telephone interview for cognitive status.

## Competing interests

The authors declare that they have no competing interests.

## Authors’ contributions

OPA conceived and designed the study, reviewed the relevant literature, registered the trial, drafted the manuscript, and will be responsible for the daily supervision of activities. CM contributed to the design of the study, designed the CBM activities, and contributed to the drafting of the manuscript. AF contributed to the design of the study and drafting of the manuscript. BG contributed to the design of the CBM activities. VH organised all assessment procedures. DG supervised the design of the internet interface of the intervention and data collection, and is responsible for the randomisation of participants. EH contributed to the design of the study. All authors read and approved the final version of the manuscript.
